# Validation of an air–liquid interface toxicological set-up using Cu, Pd, and Ag well-characterized nanostructured aggregates and spheres

**DOI:** 10.1007/s11051-016-3389-y

**Published:** 2016-03-23

**Authors:** C. R. Svensson, S. S. Ameer, L. Ludvigsson, N. Ali, A. Alhamdow, M. E. Messing, J. Pagels, A. Gudmundsson, M. Bohgard, E. Sanfins, M. Kåredal, K. Broberg, J. Rissler

**Affiliations:** Department of Design Sciences, Ergonomics and Aerosol Technology, Lund University, 221 00 Lund, Sweden; Division of Occupational and Environmental Medicine, Department of Laboratory Medicine, Lund University, 221 00 Lund, Sweden; Department of Physics, Solid State Physics, Lund University, 221 00 Lund, Sweden; Institute of Environmental Medicine, Karolinska Institutet, 171 77 Stockholm, Sweden; Institute of Emerging Diseases and Innovative Therapies (iMETI), Division of Prions and Related Diseases (SEPIA), Atomic Energy Commission (CEA), 18 Route du Panorama, 92265 Fontenay-aux-Roses, France; Chemistry, Materials and Surfaces, SP Technical Research Institute of Sweden, 223 70 Lund, Sweden

**Keywords:** Air–liquid interface, NACIVT, Cytokines, Toxicity, DMA-APM, Aggregates, SAEC, A549, Health effects

## Abstract

**Abstract:**

Systems for studying the toxicity of metal aggregates on the airways are normally not suited for evaluating the effects of individual particle characteristics. This study validates a set-up for toxicological studies of metal aggregates using an air–liquid interface approach. The set-up used a spark discharge generator capable of generating aerosol metal aggregate particles and sintered near spheres. The set-up also contained an exposure chamber, The Nano Aerosol Chamber for In Vitro Toxicity (NACIVT). The system facilitates online characterization capabilities of mass mobility, mass concentration, and number size distribution to determine the exposure. By dilution, the desired exposure level was controlled. Primary and cancerous airway cells were exposed to copper (Cu), palladium (Pd), and silver (Ag) aggregates, 50–150 nm in median diameter. The aggregates were composed of primary particles <10 nm in diameter. For Cu and Pd, an exposure of sintered aerosol particles was also produced. The doses of the particles were expressed as particle numbers, masses, and surface areas. For the Cu, Pd, and Ag aerosol particles, a range of mass surface concentrations on the air–liquid interface of 0.4–10.7, 0.9–46.6, and 0.1–1.4 µg/cm^2^, respectively, were achieved. Viability was measured by WST-1 assay, cytokines (Il-6, Il-8, TNF-a, MCP) by Luminex technology. Statistically significant effects and dose response on cytokine expression were observed for SAEC cells after exposure to Cu, Pd, or Ag particles. Also, a positive dose response was observed for SAEC viability after Cu exposure. For A549 cells, statistically significant effects on viability were observed after exposure to Cu and Pd particles. The set-up produced a stable flow of aerosol particles with an exposure and dose expressed in terms of number, mass, and surface area. Exposure-related effects on the airway cellular models could be asserted.

**Graphical Abstract:**

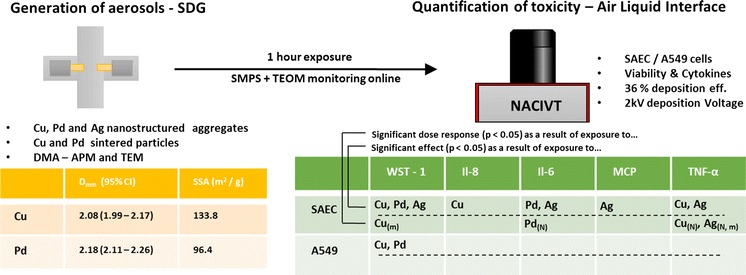

**Electronic supplementary material:**

The online version of this article (doi:10.1007/s11051-016-3389-y) contains supplementary material, which is available to authorized users.

## Introduction

- A set-up for toxicity testing of airborne metal particles was evaluated and tested
- Nanostructured aggregates of Cu, Pd, and Ag generated by spark discharge
- Dose expressed in terms of number, mass, and surface area
- Significant dose response (*p* < 0.05) for primary small airway epithelial cells (SAEC)
- Significant decrease of viability (*p* < 0.05) of A549 cells exposed to Cu and Pd

Human exposure to airborne nanostructured, metal particles occurs in many environments. Examples are industrial workplaces where metals are heated, welded, soldered, cast, and ground. Exposure to engineered nanoparticles can also take place in these environments. The particles can be near spherical in shape, created during condensation processes. The particles can be agglomerates that are the result of coagulation, and formed by a large number of primary particles bound together by weak forces. When the primary particles are fused, they are referred to as aggregates.

Submerged cell systems have been used in traditional in vitro toxicological studies, and are the basis of our knowledge of the biological effects of nanostructured particles. In these studies, the effects related to specific particle properties—such as size, shape, or surface area—can be difficult, or impossible to observe. Studies have suggested that the delivered surface area of particulates may describe their toxicity more coherently both in vitro and in vivo than mass (Donaldson et al. 2008; Waters et al. 2009).

Air–liquid interface toxicology addresses the relevance of in vitro experimentation with respect to aerosol particles. In this approach, cells are exposed directly to aerosol particles, mimicking the exposure situation in the respiratory tract. There are several air–liquid interface exposure devices, some of which are available commercially. In the Vitrocell^®^ system, no active mechanism is used to deposit the particles on the cell surface; it relies instead on natural mechanisms, such as diffusion and settling (Persoz et al. 2011). ALICE, an acronym for the air–liquid interface cell exposure system, utilizes the settling of particles on cells at the air–liquid interface (Lenz et al. 2009). The deposition is enhanced by a cloud-settling technique. The electrostatic aerosol in vitro exposure system (EAVES) uses the electrostatic deposition of particles onto cell cultures (de Bruijne et al. 2009). The nano aerosol chamber for in vitro toxicity (NACIVT) uses a static or alternating electrical field combined with unipolar charging to deposit the particles (Jeannet et al. 2014; Savi et al. 2008). The Gilling’s sampler also utilizes electrostatic precipitation onto cellular cultures at the air–liquid interface (Zavala et al. 2014).

Toxicology in the air–liquid interface has been shown to produce different results than corresponding experiments in submerged systems (using liquid dispersions of the particles). Diesel exhaust particles affect cellular cultures to a higher degree in an air–liquid interface setting, as compared to submerged experiments with what is argued as comparable doses (Holder et al. 2008). Polystyrene latex particles exert a higher degree of toxicity in the air–liquid interface as compared to submerged conditions (Fröhlich et al. 2013). Zinc oxide nanoparticles also exert toxicity at lower doses as compared to submerged conditions (Lenz et al. 2013). Silica is less toxic in an air–liquid interface setting as compared to submerged (Panas et al. 2014). It should be noted that in general dose comparability between classic in vitro and air liquid interface style is difficult.

The aim of this study is to validate a set-up for air–liquid interface toxicological research using highly characterized metal aggregate, and near spherical, aerosol particles. The set-up designed for the study represents a realization of the one first presented in Messing et al. (2012). It combines a high output aerosol particle generator with continuous online exposure monitoring. The NACIVT was for the study to ensure a high degree of deposition of aerosol particles on cell cultures, as well as to ensure a physiologically relevant environment during exposures (Jeannet et al. 2014).

Primary small airway epithelial cells (SAEC) and lung carcinoma cells (A549) were exposed at the air–liquid interface to aerosol particles of copper (Cu), palladium (Pd), or silver (Ag) aggregates or near spheres in the size range of 30–300 nm. The aggregates were characterized for size, mass, and aggregate primary structure. Cu and Ag were chosen due to their known toxicity in vitro (Foldbjerg et al. 2011; Karlsson et al. 2008), functioning as a sort of positive control. Pd was chosen due to its many uses as a catalyst in various processes, and the fact that no prior air–liquid interface research has been conducted on the material.

## Method and experimental overview

**Fig. 1:**
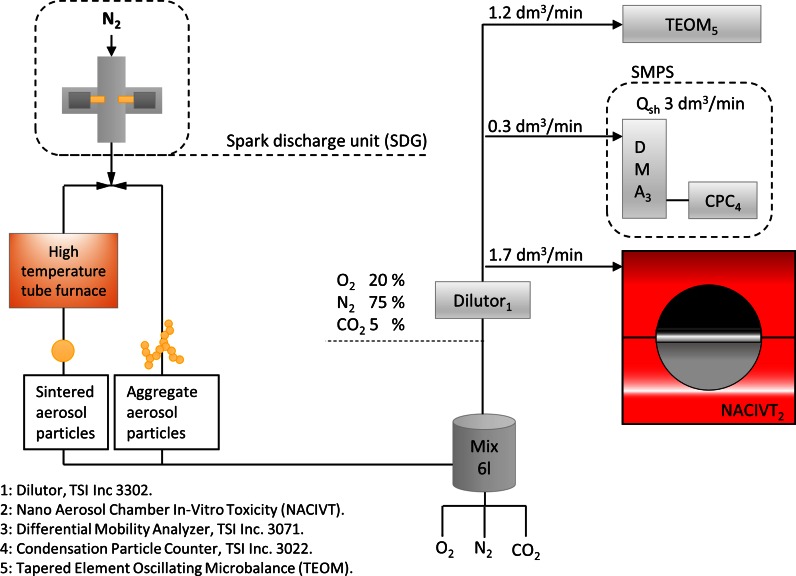
The experimental set-up for air–liquid interface exposure of SAEC and A549 cell cultures to Cu, Pd, or Ag aerosol particles. The SDG system was connected to a 6 l volume, and also fed with physiological gases. The NACIVT, SMPS, and TEOM drew aerosol particles from this volume, after being diluted to the proper exposure level, during the full 1 h exposure events. For the max. exposure levels of aerosol particles, the dilutor was bypassed from the set-up

A spark discharge system (SDG), developed at the solid-state physics Department of Lund University, was connected to a 6 l volume. The volume was, in addition to aerosol from the SDG, fed with oxygen, nitrogen, and CO_2_ to achieve a gas composed of 75 % nitrogen, 20 % oxygen, and 5 % CO_2_. Following the mixing volume, a dilutor was connected using controlling and varying the exposure dose levels. The NACIVT, scanning mobility particle sizer (SMPS) system, and tapered element oscillating microbalance (TEOM, Thermo Sci. model 1400) were connected after the dilutor, according to the set-up shown in Fig. 1. The output flow of the SDG system aerosol was controlled by the pressure in the system, and continuously monitored (controlled to near atmospheric pressure).

The set-up was used to determine the toxicological responses of SAEC and A549 cell cultures as a result of Cu, Pd, or Ag aerosol particle exposure. SAEC and A549 cultures were exposed simultaneously for a duration of 1 h, only one material per exposure. Exposures using Cu and Ag aerosol particles were performed on two occasions, indexed each as Cu_series1_, Cu_series2_, Ag_series1_, and Ag_series2_. For Pd, exposure experiments took place on only one occasion, Pd_series1_. If not specified, the particles were aggregates. For two particle types and occasions (Cu_series2_ and Pd_series1_), cell tests were performed also for spherical particles—after sintering the aggregates into spheres. Each experimental series consisted of a range of exposure levels achieved by dilution, Fig. 1. These exposure levels were indexed in the different experimental series, e.g., Cu_series2_ consist of exposures Cu_3_-Cu_5_ and sintered Cu_sint_. Sintering is a process where aerosol aggregates are compacted in a high-temperature tube furnace to near spherical shapes. Endpoints for the SAEC were WST-1 viability and the expressions of cytokines Il-8, Il-6, MCP, and TNF-α, since these cells are primary and better reflect the in vivo situation than the A549 cancer cell line. For A549, the endpoint investigated was WST-1 viability. SAEC and A549 cultures were exposed simultaneously for a duration of 1 h, only one material per exposure. An overview of the experimental matrix is shown in Table 1.

**Table 1:** The experimental matrix for determining the response with regard to viability, WST-1, for SAEC and A549 cell cultures as a result of Cu, Pd, or Ag aerosol particle exposure

	Exposure levels	Dose expression	WST-1 for SAEC/A549	Cytokines for SAEC/A549
Cu_series1_	2 (Cu_1_–Cu_2_)	*N*_*dose*_/*m*_*dose*_/*SA*_*dose*_	yes/yes	yes/no
Cu_series2_	3 (Cu_3_–Cu_5_) + 1 (Cu_sint_)	*N*_*dose*_/*m*_*dose*_/*SA*_*dose*_	yes/yes	yes/no
Pd_series1_	3 (Pd_1_–Pd_3_) + 1 (Pd_sint_)	*N*_*dose*_/*m*_*dose*_/*SA*_*dose*_	yes/yes	yes/no
Ag_series1_	3 (Ag_1_–Ag_3_)	*N*_*dose*_/*m*_*dose*_/–	no/no	yes/no
Ag_series2_	4 (Ag_4_–Ag_7_)	*N*_*dose*_/*m*_*dose*_/–	yes/yes	yes/no

For SAEC, the expression of cytokines was also quantified as a result of exposure

The cellular dose was calculated in terms of number of particles (*N*_*dose*_), mass of particles (*m*_*dose*_), and surface area of particles per square centimeter of cell surface (*SA*_*dose*_). This was achieved by combining exposure data with the deposition efficiency, *E*_*SEM*_, of the NACIVT chamber. The measurements of the particle mass-mobility relations for the calculation of mass and surface area dose were performed as separate experiments (i.e., not during actual exposures in the NACIVT).

### SDG generator

A spark discharge generator (SDG) continuously generated aerosol particles of Ag, Cu, or Pd during the exposure experiments. In short, material is evaporated from two opposing electrodes by a high-frequency spark. Electrode distance, charging current, electrical capacitance, and carrier gas flow of nitrogen gas (>99.9 % in purity) could be controlled in order to optimize particle output. When generated, the aerosol particles consist of metal aggregates (i.e., smaller primary particles fused together). The primary particles form shortly after the spark discharge from the supersaturated metal vapor and form the larger aggregates. The aggregates can optionally be sintered to near spheres by a high-temperature tube furnace (Fig. 1).

### Nano aerosol chamber for in vitro toxicity—NACIVT

The device used for the air–liquid interface in vitro exposures was the NACIVT. It combines an incubation environment, controlled relative humidity, and temperature, with high-efficiency electrostatic deposition onto the cell cultures. The NACIVT can hold up to 24 inserts of individual cell cultures for each exposure experiment (Jeannet et al. 2014).

For the experiments, the relative humidity was set to 85 % and the chamber temperature to 37 °C. The NACIVT was allowed to warm up for at least 1 h prior to any exposures in order for the relative humidity and temperature to stabilize. The insert holder was preheated in an incubator prior to exposures. For each experiment, all of the insert holder’s wells were used to avoid affecting the flow regime of the aerosol during exposures (i.e., all 24 wells were filled with either cell cultures or empty inserts). The cell cultures were provided with basal media: 500 µl for SAEC and 400 µl for A549. The A549 cell cultures also had a 40 µl apical media added during the experiments in the NACIVT. The deposition voltage during cell exposures was 2 kV non-alternating.

#### Deposition efficiency

The deposition efficiency (*E*_*SEM*_) was defined as the fraction of the particles entering the NACIVT that were deposited on the surface of the silica wafers placed in the cell inserts. Silica wafers were placed in the inserts and treated as if they had been seeded with cells for an in vitro experiment, with 400 µl liquid in the well below the insert and no apical media in the inserts. As for the cell cultures, the deposition voltage was 2 kV. The total number of particles entering the NACIVT was determined by the SMPS. The aerosol particles used for the determination of *E*_*SEM*_ were sintered Ag with a count median diameter (CMD) of 53.2 nm, and a geometric standard deviation (GSTD) of 1.55.

The SEM images were analyzed using *imageJ* software (Rasband 1997–2015). By using the *Smooth*, *Threshold*, and *Make Binary* functions, the deposited particles were separated from the wafer background. The particles were then automatically registered using the *Count Particles* function. In the process, noise consisting of clusters of 1–3 pixels was also registered in significant amounts for several images. In order to avoid this, a lower size limit was set below which no particles were considered.

Wells 6, 18, and 24 of the NACIVT were used to determine *E*_*SEM*_. For well 6, a total of five sub-areas were investigated. For each of the sub-areas, three SEM images were analyzed. For wells 18 and 24, three SEM images were analyzed.

### Scanning mobility particle sizer and tapered element oscillating microbalance

The number size distributions of the aerosol particles were measured continuously during the exposure experiments. A differential mobility analyzer (DMA, TSI Inc. 3071) was coupled with a condensation particle counter (CPC, TSI Inc. 3022) together with *AIM* software (TSI Inc.) that together constituted a scanning mobility particle sizer (SMPS). A bipolar charger was set at the DMA inlet, resulting in a known charge distribution of the aerosol particles. For the experiments, the aerosol-to-sheath airflows were set to a 1:10 ratio, 0.3–3 dm^3^/min (Fig. 1).

The mass concentration of aerosol during the exposures was measured with a tapered element oscillating microbalance (TEOM, Thermo Sci. model 1400). Aerosol is deposited onto a filter oscillating at a certain frequency. After deposition, the frequency shift is correlated to the deposited particle mass. An aerosol mass concentration, *c*_*m*-*TEOM*_, is calculated by relating mass to flow.

### The dose

Three dose measures were calculated and related to viability and cytokine expression, *N*_*dose*_, *m*_*dose*_, and *SA*_*dose*_. Dose with respect to number (N_dose_) is calculated from the SMPS. For spherical particles, the mass and surface area are also determined from the SMPS number size distribution. However, for aggregated particles, the mass-mobility relationship does not follow m∝*d*_*me*_^3^ as it does for spheres (Rissler et al. 2013). The mass and surface area of the aggregates are obtained by combining SMPS spectra with the mass-mobility relationship measured using an aerosol particle mass analyzer (APM), Sections “Mass-mobility relation—DMA-APM,” “Primary particle size and specific surface area—SSA,” and “Calculation of dose.”

#### Mass-mobility relation—DMA-APM

The aerosol particles generated were characterized using a DMA coupled in series with an aerosol particle mass analyzer (APM, Kanomax model 3600) and CPC; the system is referred to as DMA-APM. The DMA selects a particle mobility size that in turn is characterized with respect to mass by the APM. By stepping the voltage in the APM, the average mass of the individual particles of the selected distribution can be estimated (Ehara et al. 1996). The specific set-up used in the study is described in more detail by Rissler et al. (2013). The set-up was calibrated using polystyrene latex spheres of a known density (1.05 g/cm^3^) as described by McMurry et al. (2002).

For spherical particles, the mass and diameter are related according to m∝d_p_^3^. For aggregates formed by diffusion-limited cluster aggregation, the mass-mobility relationship is instead typically described by a power law (Park et al. 2003; Rissler et al. 2013):

1 $$m_{agg} (d_{me} ) = k \cdot d_{me}^{{D_{mm} }}.$$

The particle mass, *m*_*agg*_, is a function of the mobility diameter, *d*_*me*_, the K-factor, *k*, and the mass-mobility exponent, *D*_*mm*_. The power law fitted to the experimental data is expressed in terms of SI units. The characterization of size-dependent mass was performed as a separate experiment, not during the actual cell exposures, but found to be stable between experiments. The power law relation allows for the transformation of the size number distribution to mass size distribution.

#### Primary particle size and specific surface area—SSA

Samples of Cu and Pd aggregate particles were collected to determine the Sauter primary particle diameter. The Sauter diameters of the primary particles were calculated based on an analysis using *imageJ* (Rasband 1997–2015). The Sauter diameter is used to calculate the specific surface area, *SSA*_*TEM*_, of the metal aggregates. The method, and the Sauter diameter, is described in detail by Bau et al. (2010) and Svensson et al. (2015).

The collection of particle samples took place in the NACIVT, alongside actual exposure of cell cultures, onto lacey carbon-coated copper grids glued onto semiconductor silicon substrates. The samples were then visualized using transmission electron microscopy (TEM, model 3000F 300 kV, JEOL).

#### Calculation of dose

The particle number dose, *N*_*dose*_, was calculated as the product of the average total particle number concentration entering the NACIVT, *c*_*N*_, the total aerosol volume passed over the insert, *v*, the deposition efficiency *E*_*SEM*_ divided by the insert membrane surface area, S*A*_*insert*_:

2 $$N_{dose} = \frac{{c_{N} \cdot v \cdot E_{SEM} }}{{SA_{insert} }}$$

Assuming a confluent layer of cells on the insert membrane *N*_*dose*_ represents the deposited particle number per cm^2^ of cells (#/${\text{cm}}_{\text{cell}}^{ 2}$). For an experiment of 1 h exposure, *v* was 1500 cm^3^ for one insert of 6.5 mm in membrane diameter (0.33 cm^2^).

The mass dose, *m*_*dose*_, was calculated using the total aerosol particle mass concentration, *c*_*m*_, as follows:

3 $$m_{dose} = \frac{{c_{m} \cdot v \cdot E_{SEM} }}{{SA_{insert} }}$$

For Cu and Pd aggregates, the *c*_*m*_ was calculated as the sum of the mass size distribution. The mass size distribution was calculated as follows:

4 $$dm/dlogD_{P} \left( {d_{me} } \right) = dN/dlogD_{P} \left( {d_{me} } \right) \cdot m_{agg} (d_{me} )$$

The sum of the mass size distribution was calculated as

5 $$c_{m} = \mathop \sum \nolimits \frac{{dm_{agg} }}{{dlogd_{me} }} \cdot dlogd_{me}$$

For Ag aggregates, the total mass concentration, *c*_*m*_, was determined using TEOM measurements, *c*_*m*-*TEOM*_.

The surface area distributions of the Cu and Pd aggregates were obtained by multiplying Eq. 4 by the specific surface area SSA_TEM_, determined by methods described earlier:

6 $$dSA/dlogD_{P} \left( {d_{me} } \right) = dN/dlogD_{P} \left( {d_{me} } \right) \cdot m_{agg} \left( {d_{me} } \right) \cdot SSA_{TEM}$$

The total surface area concentration, *c*_*SA*_, was calculated as the sum of the surface area distribution, similar to Eq. 5. The surface area dose, *SA*_*dose*_, was calculated as

7 $$SA_{dose} = \frac{{c_{SA} \cdot v \cdot E_{SEM} }}{{SA_{insert} }}.$$

For sintered Cu and Pd aerosol particles, the *m*_*dose*_ and *SA*_*dose*_ were calculated assuming that the *d*_*me*_ was the true physical diameter. Bulk densities of 8.96 and 12.02 g/cm^3^ for Cu and Pd, respectively, were used for the calculations.

### Cell cultures and endpoints

Human primary small airway epithelial cells (SAEC; Cat no: CC-2547, Lonza, USA) and alveolar carcinoma cell line (A549) were grown in 37 °C, 5 % carbon dioxide, and 85 % relative humidity.

The primary SAEC cells were grown in the S-ALI Growth Medium (cat no: CC-3281, Lonza) with S-ALI™ SingleQuots ™ containing nutrient and antibiotics (cat no: CC-4538, Lonza). After passaging the cells two times, 50,000 cells were seeded in each 6.5 mm insert containing a 0.4 µm polystyrene membrane (Corning NY, U.S.A.) with 100 µl of S-ALI Growth Medium in the apical surface and 500 µl of S-ALI Growth Media on the basal chamber of all wells with the inserts. On the following day, the growth medium from the apical surface was removed and the growth medium in the basal surface was replaced with 500 µl of S-ALI Differentiation Medium (cat no: CC-3282, Lonza) with SingleQuots ™ containing the nutrient mixture and antibiotics and inducer to promote differentiation. This step is known as the ‘airlift’ and promotes differentiated primary airway cells to be in direct contact with the particles in a humid condition, mimicking an in vivo situation. The differentiation media from the basal chamber was changed every second day. After the airlift, cells were exposed in the air–liquid interface chamber between the 5th and 7th day.

A549 was grown in the F-12 Nutrient Mixture medium (cat no: 21765029, Invitrogen, Sweden) with 10 % heat-inactivated fetal bovine serum (FBS, cat no: 16000036, Invitrogen), 100 U/mL penicillin, and streptomycin (cat no: 15140122, Invitrogen). The day before the experiment, 50,000 A549 cells were seeded in each insert (6.5 mm, containing 0.4 µm polystyrene membrane) with the F-12 medium. The NACIVT insert holder provided 400 µl of the differentiation medium; the primary cells were exposed without apical medium, while standard A549 s were exposed with 40 µl of apical medium

The cell proliferation reagent WST-1 assay (Roche, Germany) was used to determine the cell viability, according to the instructions from the manufacturer. The cytokine expression of the interleukins, Il-6 and Il-8, the tumor necrosis factor, TNF-α, and the monocyte chemotactic protein, MCP-1, were determined in the basal medium either 24 or 48 h post exposure. The analysis was performed with a multiplexed immunoassay based on Luminex technology used on a Bio-Plex 200 Luminex instrument (Bio-Rad, Hercules, CA, U.S.A.). Samples were either undiluted or diluted two times in the cell growing medium and analyzed in triplicate. Standards were diluted in the cell medium. Magnetic bead-based assays (Bio-Rad) were used for the samples according to the instructions from the manufacturer with some minor adjustments. The samples and beads were incubated for 1 h. The calibration curves were fitted using a five-point regression model and the results were evaluated in the Bio-Plex Manager Software 6.0 (Bio-Rad).

### Statistical analysis of toxicological data

The response regarding cell viability and cytokine expression for SAEC and A549 was calculated as the ratio between exposed and control cells (Exposed/Control). The control was exposed to aerosol particle free air, 5 % CO_2_, in the NACIVT with the same conditions as for the cell culture experiments.

**Fig. 2:**
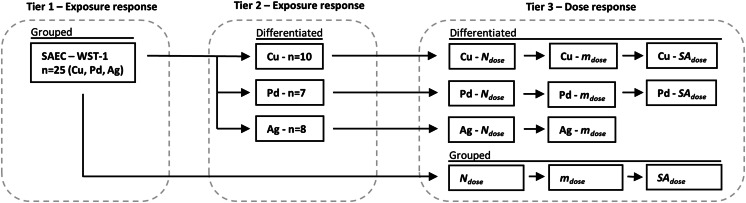
Three tiers of toxicological analysis for SAEC illustrated with respect to WST-1 viability assay. In tier 1, the data are grouped. Particle material, dose, and morphology are disregarded in the analysis. In tier 2, the data are differentiated regarding particle material, but dose and morphology are still disregarded. In tier 3, dose and morphology are also considered and a possible dose–response relation is investigated. For Cu and Ag, series 1 and 2 are pooled for all statistical analysis

The analysis of the toxicological results was performed in three tiers. The statistical analysis was also based on all the data of Cu and Ag (e.g., Cu and Ag series 1 and 2 were pooled). In the first tier, all data were grouped together, considering simply the cells as exposed or not, disregarding dose and particle material. In the second tier, the material of the particles was also taken into account, still disregarding dose and morphology. In tier 3, the dose and morphology were also included and the data were analyzed for possible dose–response relation. This analysis was performed with number, mass, and surface area-weighted dose metrics. The three tiers in the analysis are illustrated in Fig. 2 for the case of SAEC in relation to WST-1.

For tiers 1 and 2, the average exposed/control ratio was calculated together with the 95 % confidence interval. For tier 3, the dose response was analyzed using linear regression analysis, linear model. A dose response is here defined as a statistically significant (*p* < 0.05) coefficient of slope.

## Results

### Exposure response—tiers 1 and 2

**Fig. 3:**
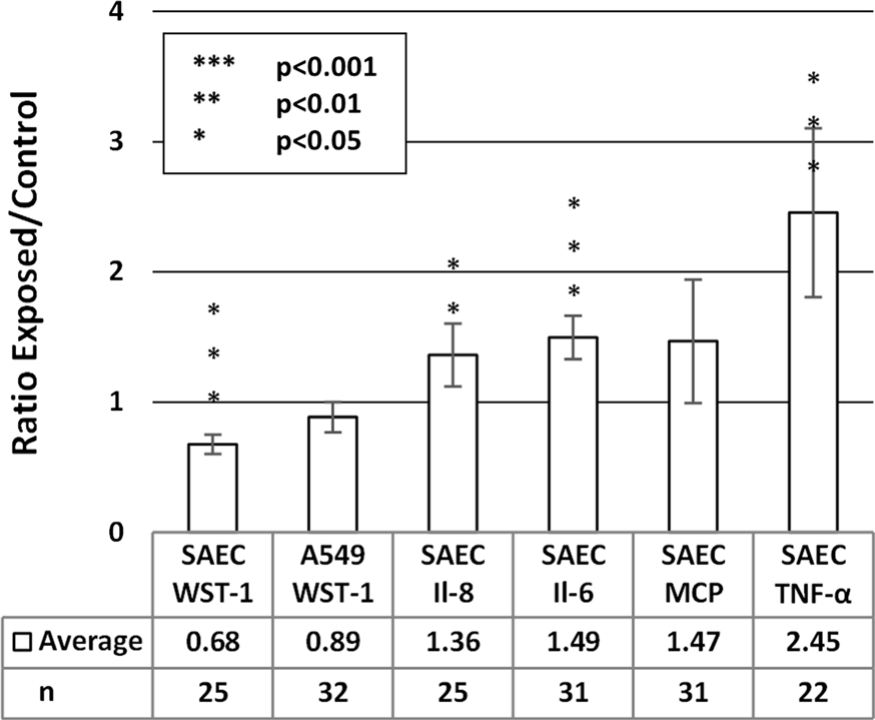
Tier 1 analysis showed that the exposure to particles reduced the viability of primary SAEC cells but increased the expression of cytokines. In the tier 1 analysis of the toxicological data, the material, shape, and the dose of the metal aggregates are not considered. The data are presented as the ratio between the exposed cells and unexposed controls. The number of *data points* is presented as *n*

In the tier 1 analysis, the cells are considered as wither exposed or unexposed, disregarding particle composition, shape, and delivered dose. In the tier 1 analysis, the SAEC showed a significant decrease (*p* < 0.001) of viability, an increase for Il-6 and TNF-α (*p* < 0.001), as well as Il-8 (*p* < 0.01) expression. For the MCP-1 expression of SAEC and WST-1 for A549, no significant response (*p* > 0.05) was observed, Fig. 3.

**Fig. 4:**
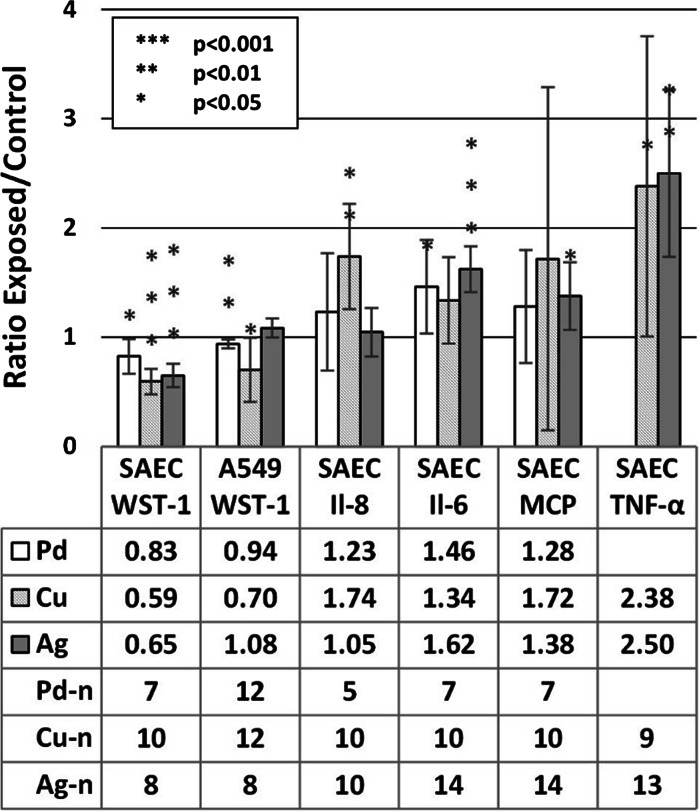
Tier 2 analysis, where the aerosol particle composition is taken into account, showed that exposure to Cu, Pd, and Ag significantly decrease viability of SAEC and had an effect on cytokine expression. The viability of A549 is also significantly reduced after exposure to Cu and Pd aerosol particles. The data are presented as the ratio between exposed and unexposed control cells. The number of *data points* is represented here as Cu–n, Pd–n, and Ag–n

In the tier 2 analysis, the data were also differentiated regarding particle material. Thus, the results listed in the columns in Fig. 3 are divided into Pd, Cu, and Ag categories in Fig. 4. Dose and shape are still disregarded.

The differentiated analysis showed that Cu, Pd, and Ag aerosol particles induced a significant decrease (*p* < 0.05) with respect to viability, WST-1 of SAEC. Exposure to Pd showed a significant increase (*p* < 0.05) of Il-6 for SAEC, while showing no significant effects on Il-8, MCP or TNF-α (below level of detection, LOD). The SAEC expression of Il-8 and TNF-α as a result of Cu exposure significantly increased (*p* < 0.01 and 0.05, respectively). Exposure to Ag induced a significant increase in the expression of Il-6, MCP, and TNF-α (*p* < 0.05) for SAEC.

While no effect was seen for the A549 cells on viability in the grouped analysis, a significant effect was observed from both Pd (*p* < 0.01) and Cu (*p* < 0.05) exposures when the data were differentiated concerning composition (Fig. 4).

### Dose response—tier 3

**Fig. 5:**
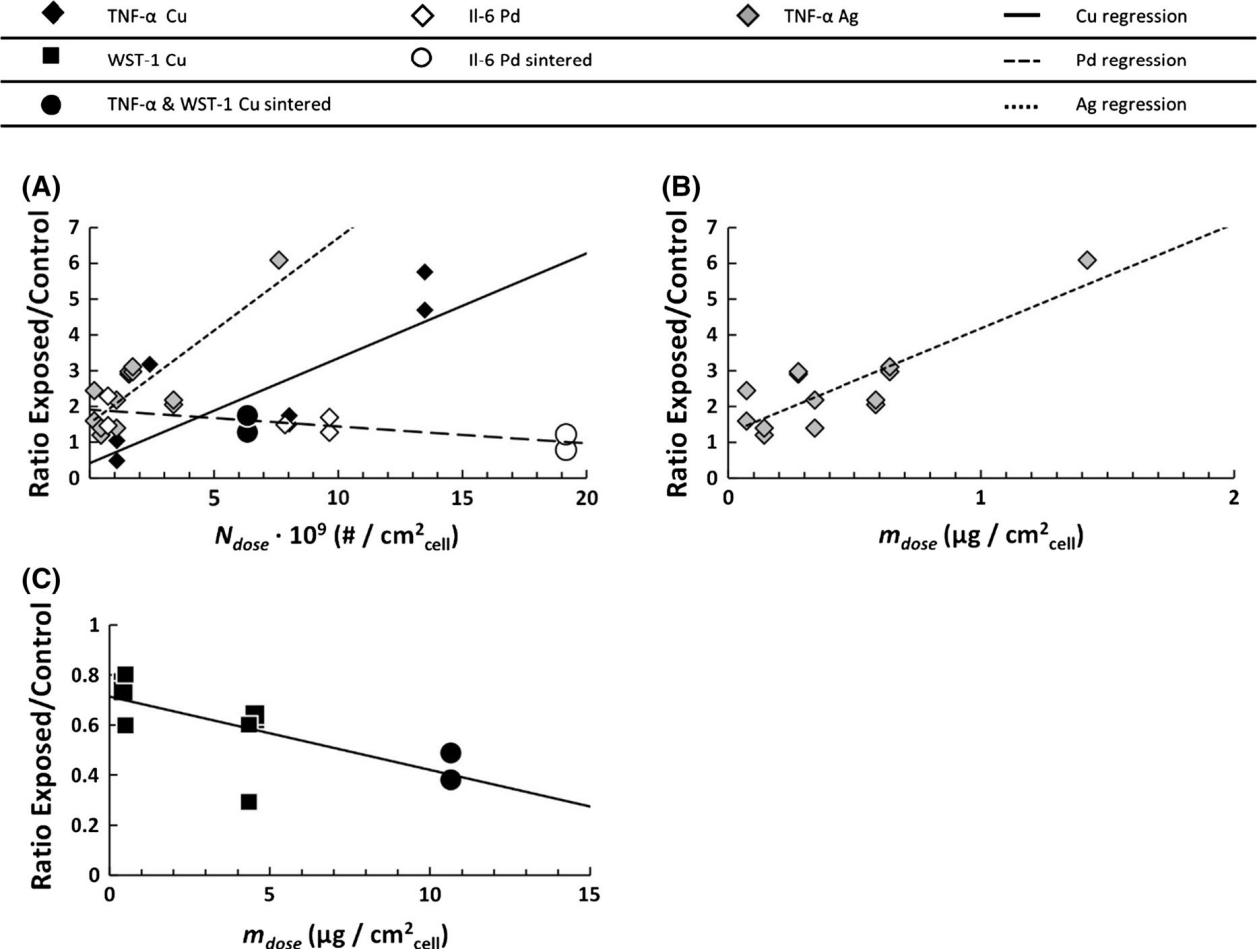
Statistically significant, *p* < 0.05, dose response for SAEC cells exposed to nanostructured metal particles, for A549, no significant dose response was observed. **a** Statistically significant, *p* < 0.05, dose response relations for TNF-α and Il-6 *endpoints* with dose expressed in terms of number, N_Dose_, after exposure to Cu, Pd, or Ag. **b** A statistically significant dose response, *p* < 0.05, was determined for SAEC expression of TNF-a exposed to Ag aerosol particles with dose expressed as mass, m_Dose_. **c** A statistically significant dose response, *p* < 0.05, was determined for SAEC viability, WST-1, after exposure to Cu aerosol particles with dose expressed as mass, m_Dose_

The final tier of the analysis relates the observed effects to the corresponding dose of aerosol particles, expressed in terms of number, mass, or surface area (Fig. 5a–c and electronic supplementary material). A linear model regression analysis was performed to determine the significance of the coefficient of slope, the dose–response relation. The analysis of dose response was performed by considering the particle material (referred to as differentiated analysis) as well as disregarding it (referred to as grouped analysis). The latter analysis is similar to the analysis in the tiers 1 and 2. The data series for Cu and Ag were pooled for the analysis, also the data points for sintered particles were included.

The statistical significance of the dose response (dose expressed in terms of particle number, *N*_*Dose*_) for the grouped data with regard to SAEC TNF-α expression was *p* = 0.004. No other significant coefficient of slope (i.e., dose response) can be asserted based on grouped analysis of either SAEC or A549 for WST-1 or cytokine expression. The complete results from the regression analysis can be found in the electronic supplementary information.

For the differentiated analysis of SAEC TNF-α expression exposed to Cu or Ag, the significance was *p* = 0.015 and 0.001, respectively, for dose response (*N*_*Dose*_). When the dose of Cu and Ag aerosol particles was expressed in terms of mass (*m*_*Dose*_), Ag showed a significant dose response (*p* = 0.0003), Fig. 5b. An ANOCOVA analysis of the TNF-α data, with material type as covariate, indicated that the dose response for Cu and Ag cannot be significantly differentiated from the common average, *p* = 0.164, when the dose was expressed in terms of particle number (*N*_*Dose*_). For Pd particles, all exposures resulted in a level of TNF-α expression below level of detection (LOD).

The differentiated analysis showed that a linear dose response, a coefficient of slope with a significance of *p* = 0.025, was asserted for SAEC viability exposed to Cu aerosol particles with a dose expressed in terms of mass (*m*_*Dose*_), Fig. 5c. Also, a coefficient of slope with a significance of *p* = 0.044, was asserted for Il-6 expressed by SAEC exposed to Pd with a dose expressed in terms of particle number (*N*_*Dose*_).

### Aerosol characteristics and dose

The aerosol characteristics regarding particle size distributions, primary particle size, aggregation state, mass-mobility relation, exposure levels, and dose are presented. The deposition efficiency determined using SEM image analysis is also presented.

#### TEM and primary particle analysis

**Fig. 6:**
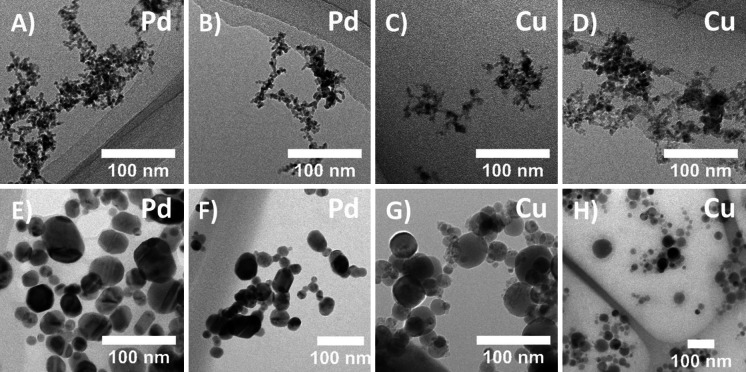
**a**–**b** Aggregates of Pd visualized using TEM. **c**–**d** Aggregates of Cu visualized using TEM. The aggregates comprised smaller fused primary particles. **e**–**f** Spherical, sintered Pd. **g**–**h** Spherical sintered Cu. The sintered particles are aggregates compacted by thermal forces shortly after generation in a high-temperature tube furnace

Aggregates of Cu and Pd were, as visualized by TEM, fractal structures composed of smaller primary particles partly fused together (Fig. 6a–d). The sintered Pd aggregates were compacted to near spherical shapes, appearing crystalline. Sintered Cu aggregates appear spherical and no apparent crystal structure can be observed (Fig. 6e–h). The TEM images used for primary particle analysis were taken from the lowest exposure level of Cu and Pd aggregates according to methodology described in Section “Primary particle size and specific surface area—SSA.”

**Fig. 7:**
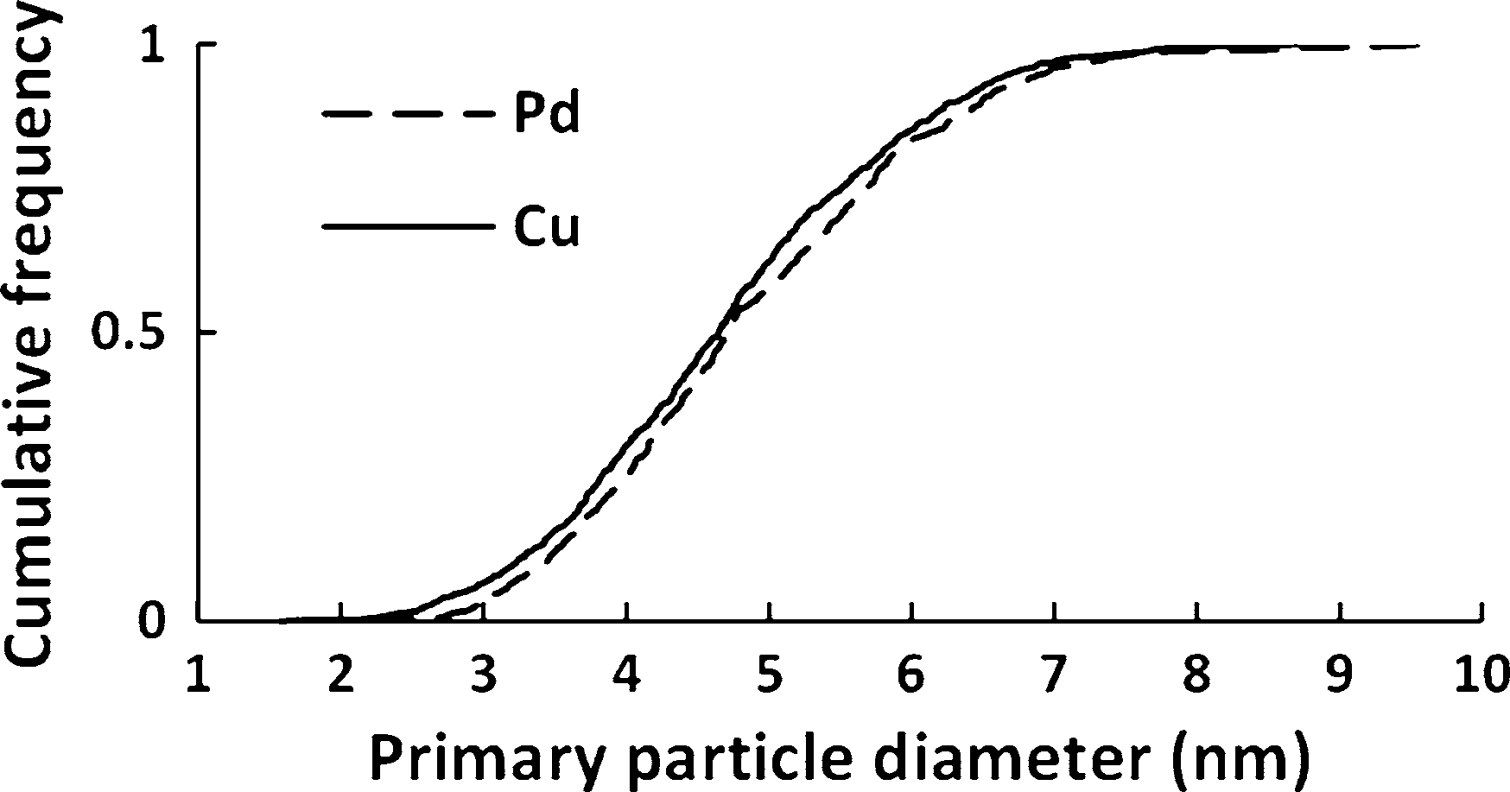
The cumulative frequency distribution of Cu and Pd primary particles from aggregates generated by the SDG

The numbers of primary particles measured for Cu and Pd aggregates were 886 and 705, respectively. The cumulative frequency distributions are shown in Fig. 7, revealing similar distributions for Cu and Pd.

The mean, *µ*, and standard deviations, *σ*, of the distributions were determined for the number, surface area, and mass-weighted distributions of the primary particles. The Sauter primary particle diameter, *d*_*va*_, and specific surface area (SSA_TEM_) were calculated using the methodology described by Svensson et al. (2015) (Table 2).

**Table 2:** The number, surface area, and mass-weighted mean and standard deviations of the Cu and Pd primary particle distributions

	*d*_*Nr*_ ± σ (nm)	*d*_*SA*_ ± σ (nm)	*d*_*m*_ ± σ (nm)	*d*_*va*_ (nm)	SSA_TEM_ (m^2^/g)
Cu	4.65 (3.51–5.92)	4.93 (3.78–6.23)	5.21 (4.04–6.50)	5.00	133.8
Pd	4.71 (3.66–6.08)	5.10 (3.92–6.44)	5.43 (4.16–7.05)	5.18	96.4

The Sauter primary particle diameter, d_va_, and SSA_TEM_ were calculated using the methodology described by Svensson et al. (2015)

The SSA_TEM_ was used for the conversion from mass to surface area distribution, as described in Section “Calculation of dose.”

#### Summarized exposure and dose

**Fig. 8:**
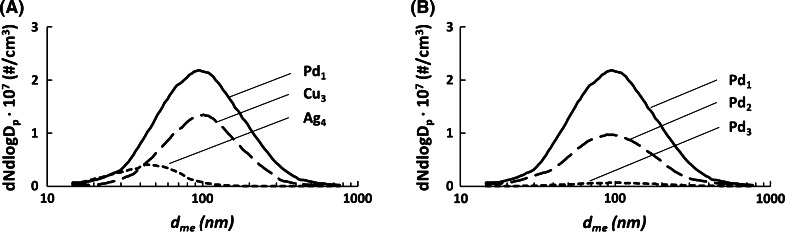
**a** Number size distributions of Cu_1_, Ag_1_, and Pd_1_ aerosols as measured online by SMPS. The *distributions* represent the maximum exposures for aggregates in this study. **b** The three exposure levels of Pd used for the study, illustrating the dilution of the Pd aerosol

The number size distributions of the aggregates and spheres were measured online during the experiments using SMPS. Number size distributions of Cu_1_, Pd_1_, and Ag_1_ are presented in Fig. 8a. The dilution was also successful and did not systematically affect the count median diameter (CMD) or geometric standard deviation (GSTD), see Fig. 8b and Table 3. Both particle number concentration, measured by SMPS, and mass concentration, measured by TEOM, indicate that the generated aerosols of metals aggregates or spheres were stable for the duration of the experiments.

**Table 3:** Aerosol characteristics, number concentration, number size distribution characteristics, mass concentration, and calculated dose levels of Cu, Pd, and Ag used for exposures in the NACIVT

	*c*_*N*_ 10^6^ (#/cm^3^)	CMD	GSTD	*c*_*m*-*TEOM*_ (mg/m^3^)	*N*_*dose*_ 10^8^ (#/${\text{cm}}_{\text{cell}}^{ 2}$)	*m*_*dose*_ (µg/${\text{cm}}_{\text{cell}}^{ 2}$)	*SA*_*dose*_ (${\text{cm}}_{\text{particle}}^{ 2}$/${\text{cm}}_{\text{cell}}^{ 2}$)
*Cu*_*series1*_							
*Cu*_*1*_	8.55 ± 1.84	87.1	1.64	1.77 ± 0.45	134.9	4.5	6.1
*Cu*_*2*_	1.50 ± 0.88	63.3	1.65	0.38 ± 0.13	24.1	0.4	0.6
*Cu*_*series2*_							
*Cu*_*3*_	7.89 ± 0.08	133.0	1.73	–	80.2	4.3	5.8
*Cu*_*4*_	2.58 ± 0.06	115.1	1.67	2.20 ± 0.08	42.0	1.7	2.2
*Cu*_*5*_	0.67 ± 0.01	118.3	1.77	0.68 ± 0.06	10.9	0.5	0.7
*Cu*_*Sint*_	3.90 ± 0.17	57.4	1.62	2.50 ± 0.19	63.5	10.6	0.7
*Pd*_*series1*_							
*Pd*_*1*_	14.82 ± 0.40	141.9	1.87	20.78 ± 1.62	96.5	10.1	9.7
*Pd*_*2*_	6.59 ± 0.71	136.7	1.84	7.15 ± 0.70	78.6	8.1	7.8
*Pd*_*3*_	0.45 ± 0.03	151.1	1.91	0.87 ± 0.09	7.4	0.9	0.9
*Pd*_*sint*_	11.83 ± 0.31	59.5	1.77	13.22 ± 0.81	191.6	46.6	2.1
*Ag*_*series1*_							
*Ag*_*1*_	4.70 ± 0.18	61.4	1.69	0.87 ± 0.10	76.1	1.4	–
*Ag*_*2*_	1.05 ± 0.03	58.8	1.68	0.39 ± 0.11	17.2	0.6	–
*Ag*_*3*_	1.00 ± 0.09	38.8	1.70	0.17 ± 0.11	15.7	0.3	–
*Ag*_*series2*_							
*Ag*_*4*_	2.06 ± 0.47	57.0	1.64	0.36 ± 0.19	33.6	0.6	–
*Ag*_*5*_	0.69 ± 0.12	61.3	1.67	0.21 ± 0.02	10.8	0.3	–
*Ag*_*6*_	0.26 ± 0.06	42.8	1.60	0.09 ± 0.01	4.4	0.1	–
*Ag*_*7*_	0.12 ± 0.04	40.2	1.61	0.04 ± 0.01	1.8	0.1	–

Each exposure level is indexed. For example, Cu_1_ refers to Cu aggregates, while Cu_sint_ to sintered aggregates. Exposures with Cu aerosol particles were performed on two occasions, Cu series 1 and 2; with Ag particles on two occasions, A_G_ series 1 and 2; but only on one occasion for Pd

From the number size distributions, the total particle number concentration, CMD, and GSTD were calculated (Table 3). In order for efficient referencing, the exposure levels were indexed numerically.

Due to short-circuiting of the unipolar charger in the NACIVT, the *c*_*N*_ and *c*_*m*-*TEOM*_ were not well correlated with the number, *N*_*dose*_, and mass dose, *m*_*dose*_, for Pd_1_ and Pd_2_. During the period for which the charger had short-circuited, an assumption of 20 % charged particles (instead of 100 %) was used based on the Boltzmann charge distribution. This means that rather than a deposition efficiency, *E*_*SEM*_, of 36 %, the resulting average deposition efficiencies were 14 % and 26 % for Pd_1_ and Pd_2_, respectively. The difference between Pd_1_ and Pd_2_ with respect to deposition efficiency is explained by a difference in when the short circuit occurred. No TEOM measurement could be obtained for Cu_3_ due to a sudden pressure change in the experimental system. The dose for Cu_3_ is also lower than can be expected based on *c*_*N*_ and *c*_*m*-*TEOM*_; this is due to a lower particle flow as a result of the pressure change.

Due to the relatively high particle concentrations in the SDG-generated aerosols, the CPC functioned largely in its photometric mode. This mode registers the total light scattering of the aerosol and calculates a particle concentration. The normal CPC operating mode registers individual light pulses from individual particles. Instead of using a CPC, an upgrade of the experimental set-up could utilize an electrometer, which measures the current in the aerosol. This would make the set-up more robust regarding high-aerosol concentrations. Alternatively, a second dilution system could be introduced, allowing dilution before both the SMPS and TEOM.

#### Mobility mass relation

**Fig. 9:**
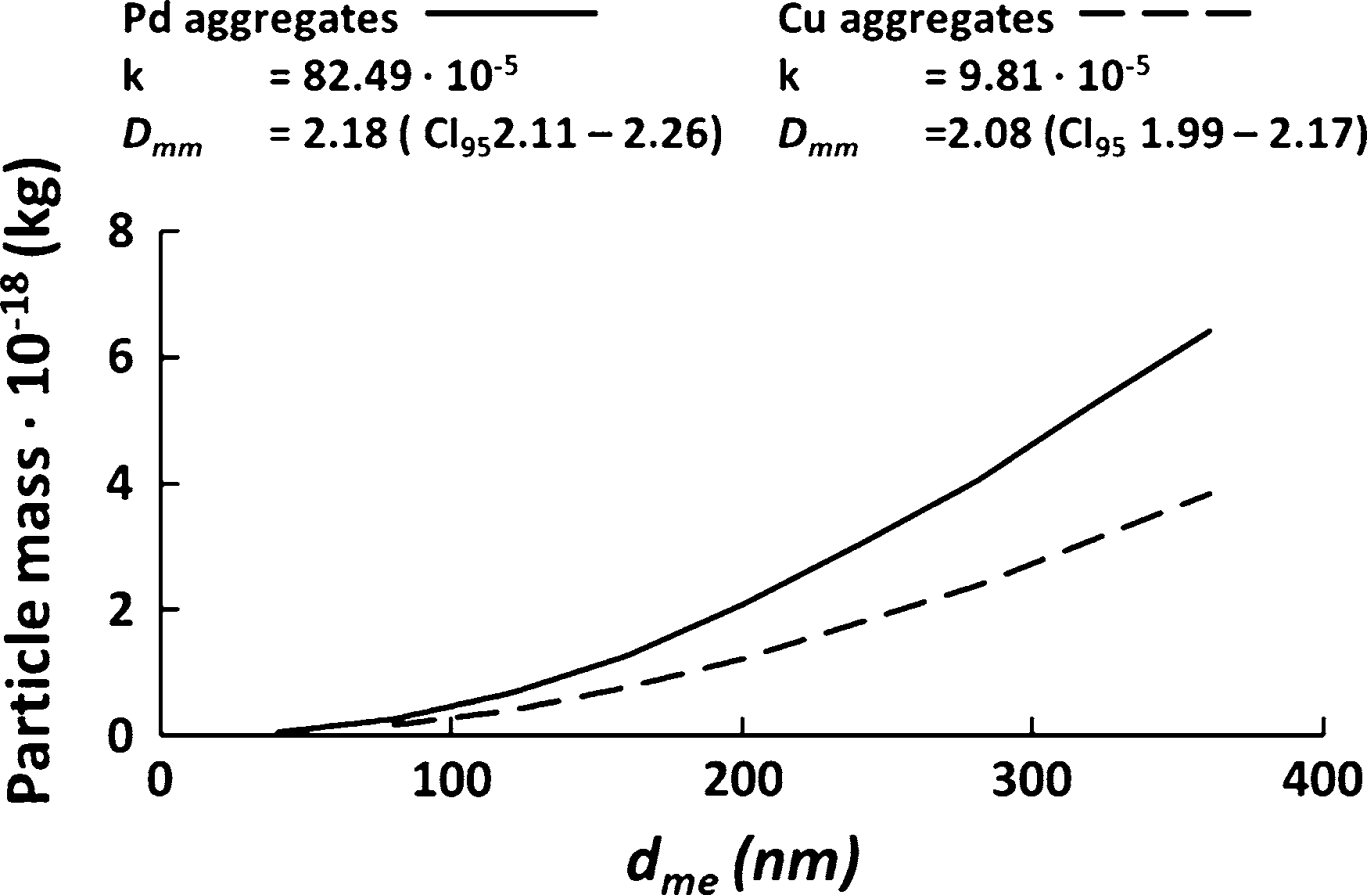
Mass-mobility relation for Cu and Pd aggregates as determined by DMA-APM

The mass-mobility relations for Cu and Pd aggregates were determined in the size range of 40–360 nm (Fig. 9).

The k-factor and mass-mobility exponent, *D*_*mm*_, were determined for the aerosol particles by fitting Eq. 1 to the mass-mobility data of the aggregates. The parameters were used to transform the number size distribution of the aggregates into mass size distribution and subsequently the delivered mass dose, see Section “Calculation of dose.” For the sintered particles, spherical particles were assumed (i.e., that the mobility diameter was equal to the geometric diameter).

#### Deposition efficiency—E_SEM_

**Fig. 10:**
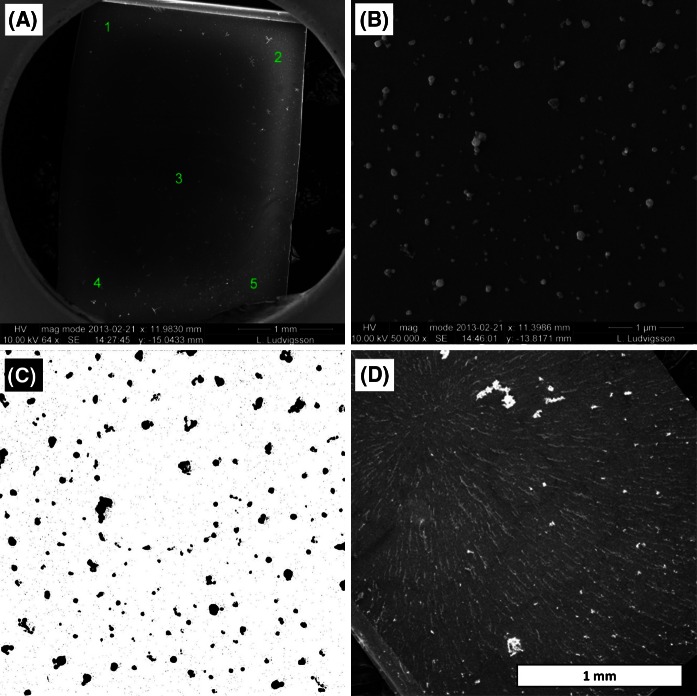
**a** Silica wafer with deposited polydisperse Ag, from well 6, for determination of deposition efficiency in the NACIVT. The analysis was performed on five areas over the wafer, each with three SEM images. **b** Sintered, spherical, polydisperse Ag aerosol deposited on silica wafer in the NACIVT. **c** Ag particles separated from the wafer background using imageJ software (Rasband 1997–2015). **d** Pd aggregates deposited on silica wafer in the NACIVT, the delivered particle amount is the highest used for cellular exposures. A deposition pattern reflection of the electromagnetic field orientation can be seen on the wafer

The deposition efficiency, using polydisperse sintered Ag aerosol particles, was determined in the three wells of the NACIVT: wells 6, 18, and 24. For well 6, three SEM images from each of five separate regions on the wafer were analyzed (Fig. 10a). SEM images from wells 18 and 24, three from each well, were chosen from the central region of the wafers.

Results from well 6 show that there is variability in the deposition pattern over the wafer surface. Wells 18 and 24 lie in the same range of efficiency as those of well 6 (Table 4).

**Table 4:** Deposition efficiency of polydisperse spherical Ag in three of the NACIVT wells

Well	Area	*E*_*SEM*_ (% ± std)
6	1	55 ± 3
	2	27 ± 3
	3	43 ± 1
	4	48 ± 2
	5	11 ± 3
18	–	17 ± 4
24	–	52 ± 8
Average		36 ± 17

Well 6, divided and analyzed in five areas, three images in each area, shows some variability in efficiency over the wafer surface. Wells 18 and 24 show a deposition efficiency in the same range as well 6

Due to noise in the images, a particle size limit was set for the analysis, post *imageJ* processing. In effect, this meant that only particles above a certain size were considered for the analysis. This detection limit is dependent on the homogeneity of the wafer background as well as the SEM magnification. The effect of the set size limit is a general underestimation of the determined deposition efficiency.

## Discussion

### Toxicological response and dose

The delivered doses of aerosol particles in this study are comparable to or higher than those of other studies, while still retaining a broad spectrum from the lowest to highest dose. No air–liquid interface toxicological research has previously been reported for Pd aggregates and spheres. Furthermore, this study represents one of a few that reports the dose in a multifaceted fashion of number, mass, and surface area. Previous air–liquid interface studies on Cu particles have a reported dose range of 0.07–100 µg/cm^2^; studies on Ag particles show a reported dose in the range of 0.005–3 µg/cm^2^. A compression of the different studies is presented in the next section.

#### Comparison of Cu and Ag exposures to other air–liquid interface studies

For primary cell cultures, this study's observation agrees with that of air–liquid interface exposure of Cu aggregates generated by spark discharge by Jing et al. (2015). The Cu aggregates by Jing et al. (2015) have a primary particle size of 5 nm and number mean diameter of 9.2 nm. Both studies observe a decrease in cellular viability for primary cell cultures with an increase in dose, expressed in terms of mass. For cytokine expression of SAEC because of Cu aerosol particle exposure, a significant dose response for TNF-α could be asserted (*p* < 0.05) by this study. However, it is clear from the tier 2 analysis that a dose of 0.4–10 µg/${\text{cm}}_{\text{cell}}^{ 2}$ Cu aerosol particles produces a statistically significant (*p* < 0.05) increase in Il-8 expression. This is comparable to that of Kim et al. (2013), where a dose of 1 µg/cm^2^ Cu, generated by nebulizing a suspension of 12 nm Cu, is associated with a fourfold to fivefold increase in Il-8 expression as compared to the control.

For cancer cell line A549, the tier 2 analysis in our study showed that exposure to Cu aerosol particles significantly reduced cellular viability. However, no significant dose response for A549 exposed to Cu aerosol particles could be asserted. This is in contrast to observations by both Jing et al. (2015) and Aufderheide et al. (2013). They reported a dose-dependent decrease in viability for A549 cell cultures. The doses of Cu achieved by Aufderheide et al. (2013), Cu aerosol with aerodynamic diameter of 0.9 µm, are higher by approximately a factor of 10, while showing a response regarding viability that is similar to the one found in our study. In Jing et al. (2015), the trend is the opposite; here, doses are approximately a factor of 10 lower than this study, but still associated with a similar decrease in viability to ours. In Elihn et al. (2013), a dose of 4.1 µg/cm^2^ Cu aerosol particles was associated with a viability of 44 %, related to the control for A549 cells. Lower doses did not induce a drop in viability. The Cu aerosol particles, by Elihn et al. (2013), were generated from dry powder with a GMD of 180 nm, primary particles approximately 100 nm.

Herzog et al. (2013) produced cellular doses of Ag aerosol of 0.03 and 0.28 µg/cm^2^, generated using a nebulizer from a suspension of <100 nm Ag nanoparticles. Triple cell co-cultures were used for the study, consisting of A549 with other epithelial-airway relevant cell types added. No significant effects on cytotoxicity, expression of Il-8, or genetic marker of Il-8 and TNF-α were observed for either dose levels of Ag on the cell cultures. Our study also found that Ag in similar doses to those of Herzog et al. (2013) does not induce any significant effects on A549 cell culture viability. However, we found that the Ag aerosol, in the tier 2 analysis, induced a significant effect regarding SAEC viability, as well as for Il-6, MCP, and TNF-α expression. In Herzog et al. (2014), a dose of 3.0 µg/cm^2^ nebulized Ag, 116 nm in diameter measured by light scattering technique, with a subsequent 24 h post exposure period was associated with what is described as a moderate response in TNF-α in A549 cell co-cultures. In Holder and Marr (2013), cultures of A549 cells were exposed to either polydisperse or monodisperse sizes of Ag aerosols generated by an atomizer; the monodisperse fractions were selected using a DMA. The delivered dose of the polydisperse Ag aerosol was 0.7 µg/cm^2^ and did not have any significant effects on viability, cytotoxicity, or Il-8 expression. The size-selected fractions of Ag aerosol were 50, 75, and 100 nm. For these exposures, the dose was expressed in terms of number, mass, and surface area. Although lower doses for the size-selected aerosols were in the range of 5–26 ng/cm^2^, a statistically significant decrease in viability was observed. Translated into a number-weighted dose, for the size-selected fractions, it was in the range of 4.7–7.6 10^6^ #/cm^2^, which is approximately a factor of 100 below doses in this study.

With regard to the Ag exposures in this study, the lack of primary particle analysis in the calculation of surface area dose was not possible, according to methodology described by Svensson et al. (2015).

#### Relation to toxicological studies performed in suspension and a comment on solubility

A comparison of air–liquid interface dose levels and of liquid suspension is difficult. The theoretical tools described by Teeguarden et al. (2007) can translate a dose expressed in terms of units/cm^3^ (volume) to that of units/cm^2^ (surface). A possible first-order translation of the doses in this study in order to compare them with other studies on toxicology suspension would be to simply add a length dimension to the dose, as was done by Karlsson et al. (2009). The dose of Cu_1_ would be 4.52 µg/cm^3^ (µg/cm^3^) rather than µg/cm^2^. A physical interpretation could be that all particles in 1 cm^3^ solution sediment onto a surface of 1 cm^2^. The results from Karlsson et al. (2009) show that doses of 40 and 20 µg/cm^2^ Cu nanoparticles, 200 nm in solution measured by light scattering, are associated with a drop in viable A549 cells to <10 %, compared to control, for both doses. In Lanone et al. (2009), a reduction in viability of A549 and THP-1 as a result of Cu particle exposure was observed in a dose-dependent manner in the range of 1–100 µg/cm^3^. The dose of Cu used in Karlsson et al. (2009) was higher than any achieved in this study and also associated with larger viability effects. Given the translation of dose previously discussed, Lanone et al.'s (2009) results are comparable to ours, where a significant response was found for both A549 and SAEC in the grouped analysis for viability. For Ag exposure in solution, Foldbjerg et al. (2011) found adverse and dose-dependent decreases in A549 viability as a result of Ag exposure, ~150 nm in diameter measured by light scattering technique, in the dose range of 5–15 µg/cm^3^. Beer et al. (2012) showed that a dose of 0.5–2 µg/cm^3^ Ag, 17–70 nm by TEM measurements, was associated with a dose-dependent decrease in A549 viability; our study found no significant effects on viability for A549 in either the grouped or differentiated analysis. Beer et al. also found that the effects of silver ions on the toxicity of Ag and Cu particles could not be neglected. Some research efforts have been devoted to the toxicology of Pd in suspension. Petrarca et al. (2014) showed that a dose of 10–100 µg/cm^3^ was associated with a dose-dependent drop in viability, down to <10 % for the highest dose. The effect is also dependent on the post exposure incubation time, and the ionic Pd component is important.

Ideally, in the field of air–liquid interface research, the question of solubility would pose less of a problem in comparison to solution toxicology. This is because the particles would encounter the cell cultures and their mucosa in the same fashion as that of an in vivo situation. This is the case for the SAEC cultures of this study. However, as described in the methodology section for the A549, a small liquid volume was added on the apical side of the insert during exposure. Without this apical medium, it is possible that a more clear response in A549 viability could be observed in this study. It should also be noted that real lung cells are in motion, stretching, and shrinking, as we breathe. This can lead to a higher degree of particle movement through the mucosa to the cell surface compared to the static inserts used in vitro.

### Exposure characteristics and online monitoring

If not essential, the online characterization of exposure is highly desirable. This study used SMPS for measurements of number size distribution and total particle number concentration. The SMPS is a reliable and well-known system for measurements of sub-micrometer aerosols, and is used for exposure monitoring and calculation of dose. SMPS systems have been used successfully for several air–liquid interface toxicological studies (Elihn et al. 2013; Holder and Marr 2013; Jing et al. 2015).

A TEOM was also used to get a direct online measurement of the aerosol mass concentration. The total particle number concentrations measured correlated well with the mass concentrations from the TEOM, taking into count the varying CMDs of the distributions and the particle effective densities. The TEOM is, however, very sensitive to pressure changes as well as sudden changes in concentration. Thus, the use of several methods of exposure characterization makes the set-up as a whole less sensitive.

### Mass-mobility relation and morphology

The mass-mobility exponent, *D*_*mm*_, of the Cu and Pd aerosols is in the size range that can be expected of fractal agglomerated/aggregated metal particles formed by diffusion-limited cluster aggregation, ~2.0–2.2 (Charvet et al. 2014; Shin et al. 2009; Svensson et al. 2015). The TEM image analysis confirms the aggregate shape, and reveals that the primary particles were partly fused together. The *D*_*mm*_ for Cu and Pd is not significantly different (*p* < 0.05), indicating that the methodology did not discern any structural differences. The primary particle sizes that were determined were similar (Table 2), which also indicates a similar aggregate morphology. The sintered aggregates not only appear to be near spherical, as visualized by TEM (Fig. 6), but also show that there is a tendency toward aggregation after sintering. Whether this is due to an effect of the deposition or aggregation in air form is unclear.

The particle morphology observed in this study is not unique to particles generated by spark discharge, but can be observed as a result of other industrial and natural processes. Aggregated particles, in the size range of a 10–100 nm, can be generated by a high-temperature furnace (Elihn et al. 2013; Shin et al. 2010; Svensson et al. 2015). Sub-micrometer particles from welding processes are also known to be aggregates of smaller primary particles (Isaxon et al. 2013), as well as soot- and combustion-type particles (Rissler et al. 2013; Xiong and Friedlander 2001).

### Deposition efficiency

Knowledge of the deposition efficiency of the NACIVT is a key for determining the dose of Cu, Pd, or Ag aerosol particles. In Jeannet et al. (2014), the deposition efficiency of the NACIVT was determined using a combination of experiments and theory. In that study, 200 nm PSL and 20 nm Ag spheres were deposited on substrates after which image analyses were performed. The deposited number of spheres per surface area was related to the aerosol concentrations and flow rates, and the deposition efficiencies were calculated empirically. The size-dependent deposition efficiency was calculated, and the empirical data were used to scale the calculations of deposition efficiencies. Below 100 nm, the efficiency ranged from 20–40 % in a size-dependent manner that exponentially increased as size decreased. An image analysis also showed that more particles were deposited in the center of the insert membrane than on the edges, indicating a radial deposition pattern.

This study employed a similar methodology to that of Jeannet et al. (2014) but only using empirical determination of deposition efficiency. Sintered Ag aerosol particles, 53.2 CMD and 1.55 GSTD, were deposited onto silicon wafers with subsequent image analysis. Results from this study are in agreement with Jeannet et al. (2014), showing a deposition efficiency of on average 36 ± 17 %. Our estimation of deposition efficiency is in the upper range of that observed by Jeannet and co-authors, which can be explained by the aerosol used in our determination being in the lower size range compared to Jeannet et al. (2014). The main variance of the deposition efficiency determined can be explained by the variance observed on the different areas of well 6. Within the same area of the wafers, the analysis showed significantly less variance. However, due to the shape of the silicon wafer and placement in the insert, an evaluation of the spatial deposition pattern could not be performed in this study.

Determination of the deposition efficiency directly for aggregates of Cu and Pd was also investigated. Tests were performed collecting aggregates of Cu and Pd on silica wafers in parallel with the cell exposures. By studying the deposition of the aggregates, it became clear that these were aligned in the electric field (Fig. 10d). This resulted in the formation of an overlay of particles, ridge, and cluster formations. The results indicate that the Pd aggregates were more prone than Cu to form larger networks and clusters on the silica wafers, while Cu appeared more evenly dispersed. Both sintered Cu and Pd appeared as aggregates rather than individual particles. This makes the determination of deposition efficiency by counting single particles problematic. Furthermore, trace of the condensation of liquid was observed on several wafers during analysis. In the case of the polydisperse spherical Ag that was deposited for efficiency determination, none of the two mentioned effects were observed.

Other air–liquid interface toxicological studies have used chemical or spectroscopic methods as an alternative to image analysis, sometimes in combination with SMPS. The deposition efficiency was determined by Elihn et al. (2013) using a combination of SMPS and atomic absorption spectroscopy (AAS). Kim et al. (2013) used a nebulizer to generate an aerosol with a florescent component. The aerosol was sampled at the system’s inlet and at the site where the cell cultures were placed during exposure. The deposition efficiency was determined by relating the two concentrations at the inlet and at the cell culture using fluorescent intensity measurements. Holder and Marr (2013) used a similar method with florescent monodisperse particles.

#### Deposition efficiencies for other air–liquid interface devices

Different air–liquid interface exposure systems will have different deposition efficiencies, depending on their design and functionality. The NACIVT utilizes an electrical field to ensure a high-deposition efficiency. This study used a 2 kV alternating field achieving a 36 % deposition efficiency, which is similar to that found in Jeannet et al. (2014) for the same system, with a constant field strength and particle size range. The electrostatic aerosol in vitro exposure system (EAVES) reports a deposition efficiency for 200 and 500 nm PSL spheres of 35 and 47 %, respectively (de Bruijne et al. 2009). Since deposition efficiency is expected to drop with the mobility diameter of the particles, the reported efficiencies for the EAVES system should be interpreted as higher than that of the NACIVT. This is to some degree in contrast to the NACIVT, where efficiency is reported to drop as size increases: for 500 nm, the deposition efficiency is below 15 % (Jeannet et al. 2014). The higher efficiency of the EAVES is most likely explained by a combination of design and higher deposition field strength. Another electrostatic device is the electrostatic particulate dosage and exposure system (EPDExS). This system is characterized with regard to deposition efficiency for several particle sizes and deposition voltages, 50–530 nm and 0–10 kV, respectively. For 2 kV deposition voltage in the EPDExS, which was used in this study in the NACIVT, 100 and 200 nm particles had a deposition efficiency of >95 and 55–60 %, respectively. The EPDExS, together with the EAVES, have a higher deposition efficiency for particles over 100–200 nm compared to the NACIVT. Again, this may be due to a difference in design in the exposure chambers: Both the EAVES and the EPDExS place the cell cultures closer to the aerosol inlet than the NACIVT. This is certain to affect the measured deposition efficiency as losses in the exposure chambers are dependent on both geometry and particle residence time before arriving at the site of intended deposition.

There are also air–liquid exposure devices that use mechanisms other than electrostatic deposition. The air–liquid interface cell exposure system (ALICE) that uses a cloud droplet formation and sedimentation to ensure deposition of particles has reported deposition efficiencies from 7 to 47–50 % (Herzog et al. 2013; Lenz et al. 2009). The Vitrocell^®^ system has been used in several air–liquid interface toxicological studies. The most common version of the system relies on sedimentation and diffusion for particle deposition. In Xie et al. (2012), a Vitrocell^®^ module was built into an in-house system where cells were exposed to nebulized ZnO. No clear deposition efficiency was reported in the study, making comparisons with the literature difficult. In Kooter et al. (2013), a deposition efficiency for 50 nm particles, based on modeling rather than experiments, was reported as ~2 % for the Vitrocell^®^. The CULTEX RFS reports a size-dependent deposition efficiency over 70 % for particles in the size range from 100 nm to 10 µm. For aerosol particles below 100 nm, the efficiency decreases in a size-dependent manner to approximately 30 % (Aufderheide et al. 2013).

## Summary

The performance and functioning of an experimental set-up for toxicological studies of airborne metal aggregates using an air–liquid interface approach were tested. The airborne particles were produced by a spark discharge generator, and several techniques were used for characterizing the particles both online and offline. Primary and cancer airway cells (SAEC and A549) were exposed in a nano aerosol chamber for in vitro toxicity (NACIVT).

The exposure to the NACIVT with regard to the number size distribution, total particle number, and mass concentration showed that the flow of aerosol particles was stable during the course of the 1-hour exposure period at high enough outputs to generate a measureable toxic response. Using the DMA-APM, the mass-mobility exponents (*D*_*mm*_) of 2.08 (CI 95 % 1.99–2.17) and 2.18 (CI 95 % 2.11–2.26) for Cu and Pd aggregates, respectively, could be determined. Typical values for aggregates were formed by diffusion-limited cluster aggregation. Based on the primary particle analysis, the Cu and Pd aggregates generated for this study had specific surface areas of 134 and 96 m^2^/g, respectively. The deposition efficiency of the NACIVT was found to be on average 36 % for 53 nm CMD-sintered Ag aerosol, determined by SEM. This results are comparable with those reported for the NACIVT in Savi et al. (2008) and Jeannet et al. (2014).

By combining the deposition efficiency, particle number size distributions, mass-mobility relationship (or particle effective density) and specific surface area, number, mass and surface area based doses of aggregates and sintered particles were determined. For Cu aerosol, a dose range of 0.4–10 µg/${\text{cm}}_{\text{cell}}^{ 2}$ and for Pd, a range of 0.9–46 µg/${\text{cm}}_{\text{cell}}^{ 2}$ could be calculated. The Ag dose was in the range of 0.1–1.4 µg/${\text{cm}}_{\text{cell}}^{ 2}$ range.

Results from the tier 1 analysis showed that A549 cells were not significantly affected regarding viability, whereas a significant effect was observed for the primary SAEC viability as well as for cytokine expression. The tier 2 analysis, also considering particle material, showed that both Cu and Pd had significant effects on A549 viability and that all materials significantly affected SAEC viability. SAEC also showed altered expression of cytokines following particle exposure, particularly to Cu and Ag and to a less extent to Pd. Lastly, the tier 3 analysis identified dose–response relationships for some particles and cytokine expression in SAEC. The tiers 1–3 analyses clearly demonstrate the need to evaluate particle characteristics in relation to toxicity. Moreover, our results suggest that the primary cells are more sensitive to particles exposure than the cancer cells, which implicates that studies using A549 as a cell model for airway cells actually might underestimate the toxicity of particles. Further studies on particle toxicity in primary cells and in relation to aerosol particle characteristics are thus warranted.

The results show that the set-up described in this study is well suited for its aim to relate a multifaceted dose to a relevant cellular response of cell cultures as a result of a highly characterized exposure to nanostructured aerosols at the air–liquid interface. The characterization regime employed in the set-up allows for good comparability with other and future toxicological studies.

## Data sharing through SND

The data that this study is based upon are publicly available through Swedish National Data Service (http://www.SND.gu.se/en) by doi: 10.5878/002761. The data include primary particle analysis, dose response, mass mobility, and deposition efficiency.

It is the hope of the authors that the data will be a valuable asset and live on in research to come.

## Electronic supplementary material

Below is the link to the electronic supplementary material.

Supplementary material 1 (DOCX 688 kb)
