# Supplementary material for: Validation of an air–liquid interface toxicological set-up using Cu, Pd, and Ag well-characterized nanostructured aggregates and spheres
Source: J Nanopart Res. 2016 Mar 23;18:86. doi: 10.1007/s11051-016-3389-y (PMC4805710; doi:10.1007/s11051-016-3389-y)
Supplement: Supplementary file 1 — Supplementary material 1 (DOCX 688 kb) [file 11051_2016_3389_MOESM1_ESM.docx]

# Complete Dose-Response

A linear model regression analysis was performed to determine the significance of the coefficient of slope, the dose-response relation. The analysis of dose response was performed by considering the particle material and shape (referred to as differentiated analysis) as well as disregarding it (referred to as grouped analysis). The latter analysis is similar to the analysis in the tiers 1 and 2. The data series for Cu and Ag were pooled for the analysis, also the sintered data points were included.

Figure 1A-F show all dose response data of SAEC and A549 with regards to viability as a result of Cu, Pd or Ag aerosol particle exposure.


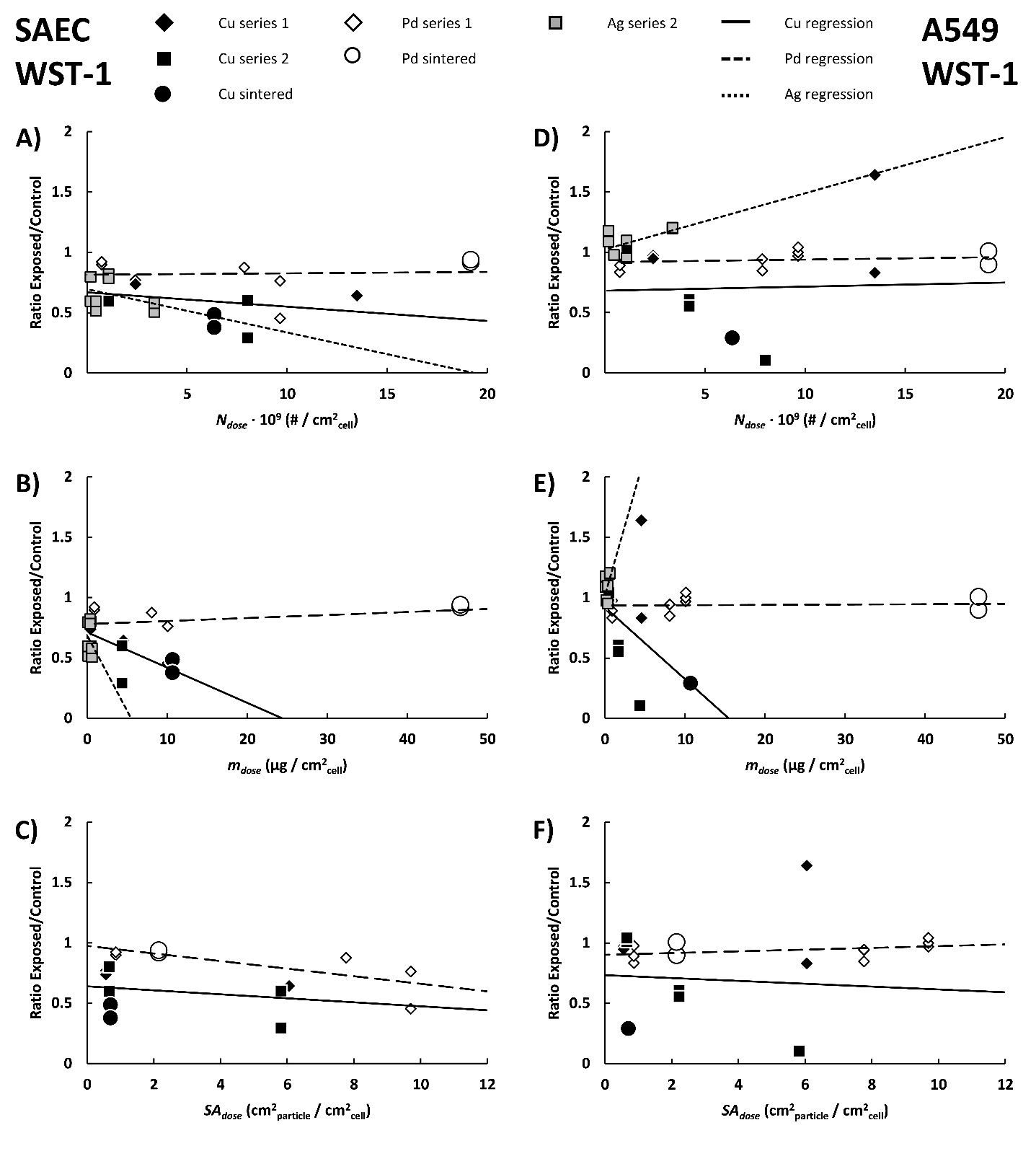


Figure 1. Tier 3 analysis showed a significant dose response for SAEC viability, decreasing with dose, for Cu exposure (dose expressed in terms of mass). A) Viability, WST-1, of SAEC related to the dose of Cu, Pd and Ag aerosol particles with dose expressed in terms of particle number / cm^2^_cell_. B-C) Instead of particle number, the viability of the SAEC is related to the mass and surface area dose of the aerosol particles. D) Viability, WST-1, of A549 related to the dose of Cu, Pd and Ag aerosol particles with dose expressed in terms of particle number / cm^2^_cell_. E-F) Instead of particle number, the viability of the A549 is related to the mass and surface area dose of the aerosol particles. For the Ag aggregates, no surface area dose was calculated for either SAEC or A549.

Table 1 summarize the p-value of the coefficient of slope for linear regression analysis of the data.

Table 1. Significance levels of the dose response (coefficients of slope) for the exposure data. The data are analyzed both as grouped and differentiated according to particle material. For example, the p-value of 0.683 is calculated for WST-1 with number-weighted dose, considering only the particle dose and not material. The data is then divided into the three material groups. Surface area (SA_dose_) weighted dose is only calculated for Pd and Cu.

|  | p-Grouped | p-Differentiated | p-Grouped | p-Differentiated |
| --- | --- | --- | --- | --- |
|  | WST-1, SAEC (*N_dose_*/*m_dose_*/*SA_dose_*) | | WST-1-A549 (*N_dose_*/*m_dose_*/*SA_dose_*) | |
| Pd | 0.683 / 0.132 / 0.392 | 0.914 / 0.531 / 0.054 | 0.786 / 0.833 / 0.504 | 0.944 / 0.809 / 0.170 |
| Cu |  | 0.348 / 0.025* / 0.442 |  | 0.923 / 0.116 / 0.844 |
| Ag |  | 0.366 / 0.626 / - |  | 0.119 / 0.237 / - |
|  | Il-8, SAEC (*N_dose_*/*m_dose_*/*SA_dose_*) | | Il-6, SAEC (*N_dose_*/*m_dose_*/*SA_dose_*) | |
| Pd | 0.817 / 0.554 / 0.990 | 0.158 / 0.204 / 0.368 | 0.188 / 0.062 / 0.817 | 0.044* / 0.063 / 0.913 |
| Cu |  | 0.895 / 0.683 / 0.690 |  | 0.493 / 0.669 / 0.803 |
| Ag |  | 0.445 / 0.409 / - |  | 0.917 / 0.770 / - |
|  | MCP-1, SAEC (*N_dose_*/*m_dose_*/*SA_dose_*) | | TNF-α, SAEC (*N_dose_*/*m_dose_*/*SA_dose_*) | |
| Pd | 0.164 / 0.221 / 0.381 | 0.060 / 0.060 / 0.901 | 0.004 / 0.945 / - | LOD |
| Cu |  | 0.368 / 0.168 / 0.341 |  | 0.015 / 0.978 / - |
| Ag |  | 0.188 / 0.097 / - |  | 0.001*** / 0.0003*** / - |

Figure 2 show SAEC expression of cytokines as a result of Cu, Pd or Ag aerosol particle expsoure, significant dose response can be seen in table 1.


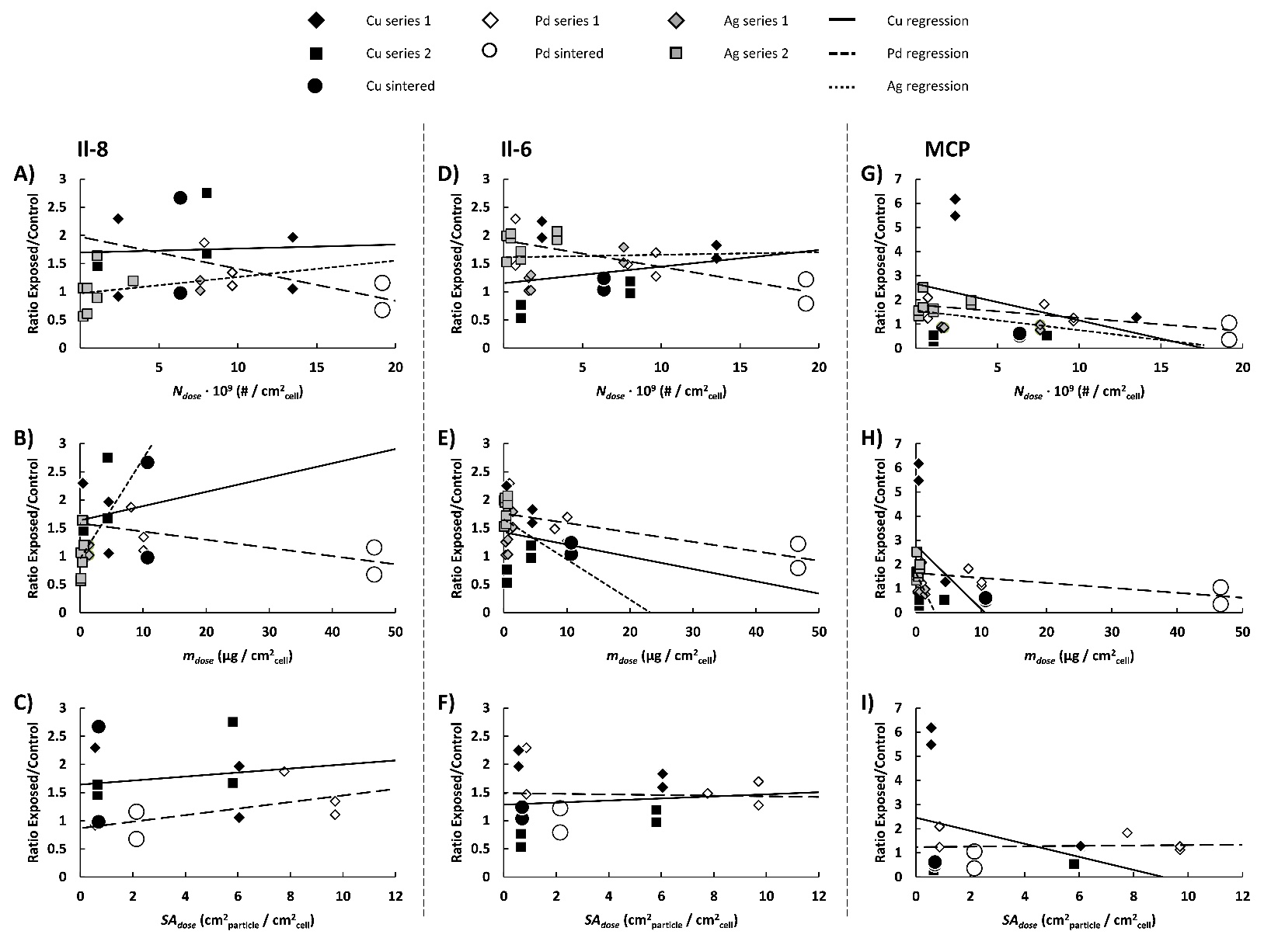


Figure 2. Expression of cytokines A-C) Il-8, D-F) Il-6 and G-I) MCP versus the delivered dose of Cu, Ag and Pd aggregates and spheres expresssed as number, mass and surface area. No significant coefficient of slope, dose response, for the grouped analysis was determined for any of the cytokines or variation of dose expresssion. A significant dose response (p=0.044) was determined for SAEC expression of Il-6 exposed to Pd aerosol with the dose expressed in terms of number. Cu and Ag series 1 and 2 were pooled for the analysis.

SAEC expression of TNF-α can be seen in Figure 3, significant dose response in Table 1.


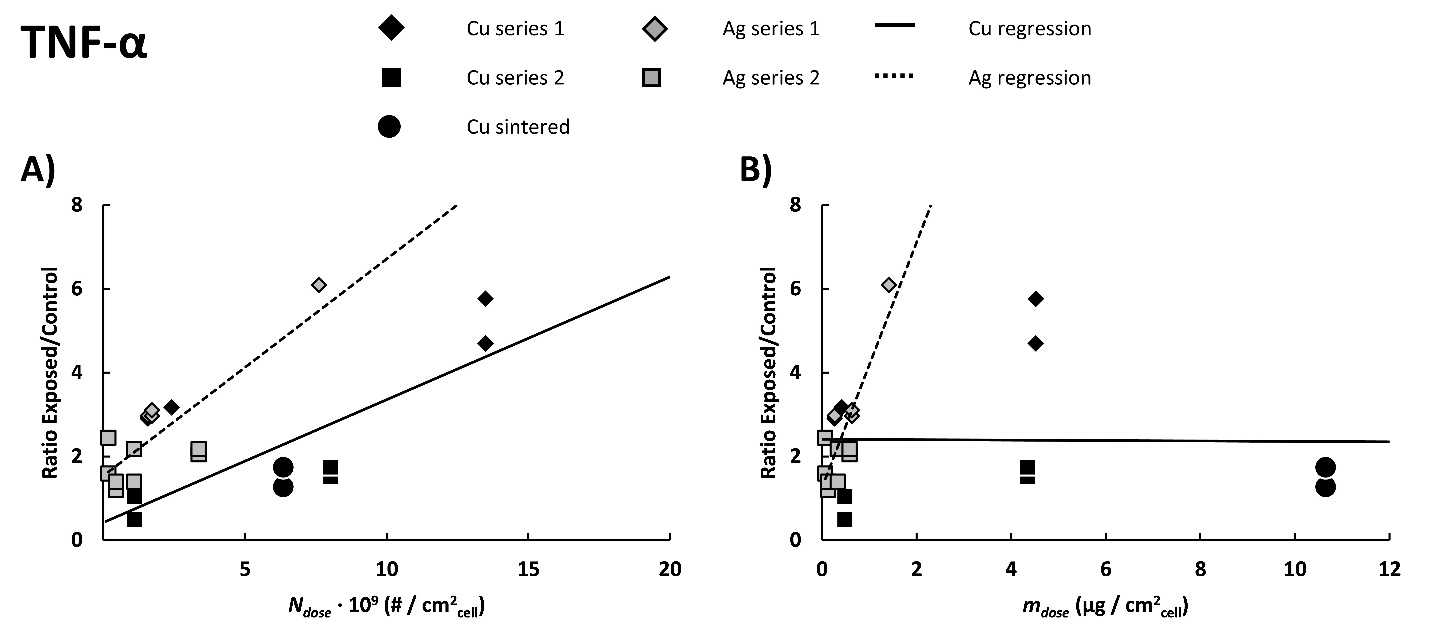


Figure 3. Expression of TNF-α from SAEC as a result of Cu and Ag aerosol exposure. A) A significant dose response can be asserted for both Cu (p=0.015) and Ag (p=0.001) with the doses expressed in terms of particle; the grouped analysis also shows a significant dose response (p=0.004). B) When the Cu and Ag aerosol particle doses are expressed in terms of mass / cm^2^ , only Ag aggregates show a significant dose response (p=0.0003); the grouped analysis does not show a significant dose response. Cu and Ag series 1 and 2 are pooled for the analysis. Exposure to Pd resulted in a TNF-α response below level of detection.
